# Bio-conjugation of antioxidant peptide on surface-modified gold nanoparticles: a novel approach to enhance the radical scavenging property in cancer cell

**DOI:** 10.1186/s12645-016-0013-x

**Published:** 2016-02-09

**Authors:** Sushma Kalmodia, Suryanarayanan Vandhana, B. R. Tejaswini Rama, Balasubramanyam Jayashree, T. Sreenivasan Seethalakshmi, Vetrivel Umashankar, Wenrong Yang, Colin J. Barrow, Subramanian Krishnakumar, Sailaja V. Elchuri

**Affiliations:** Department of Nano-biotechnology, Vision Research Foundation, Sankara Nethralaya, 18, College Road, Nungambakkam, Chennai, 600 006 India; Centre for Chemistry and Biotechnology, Deakin University, Geelong campus, Geelong, VIC 3216 Australia; Center for Bioinformatics, Vision Research Foundation, Sankara Nethralaya, 18, College Road, Nungambakkam, Chennai, 600 006 India

**Keywords:** Antioxidant peptide, Conjugate, Cancer cell, Gold nanoparticles (GNPs), Oxidative stress, Reactive oxygen species (ROS)

## Abstract

**Background:**

Functionalized gold nanoparticles are emerging as a promising nanocarrier for target specific delivery of the therapeutic molecules in a cancer cell, as a result it targeted selectively to the cancer cell and minimized the off-target effect. The functionalized nanomaterial (bio conjugate) brings novel functional properties, for example, the high payload of anticancer, antioxidant molecules and selective targeting of the cancer molecular markers. The current study reported the synthesis of multifunctional bioconjugate (GNPs-Pep-A) to target the cancer cell.

**Methods:**

The GNPs-Pep-A conjugate was prepared by functionalization of GNPs with peptide-A (Pro-His-Cys-Lys-Arg-Met; Pep-A) using thioctic acid as a linker molecule. The GNPs-Pep-A was characterized and functional efficacy was tested using Retinoblastoma (RB) cancer model in vitro.

**Results:**

The GNPs-Pep-A target the reactive oxygen species (ROS) in RB, Y79, cancer cell more effectively, and bring down the ROS up to 70 % relative to control (untreated cells) in vitro. On the other hand, Pep-A and GNPs showed 40 and 9 % reductions in ROS, respectively, compared to control. The effectiveness of bioconjugate indicates the synergistic effect, due to the coexistence of both organic (Pep-A) and inorganic phase (GNPs) in novel GNPs-Pep-A functional material. In addition to this, it modulates the mRNA expression of antioxidant genes glutathione peroxidase (GPX), superoxide dismutase (SOD) and catalase (CAT) by two–threefolds as observed.

**Conclusions:**

The effects of GNPs-Pep-A on ROS reduction and regulation of antioxidant genes confirmed that *Vitis vinifera L*. polyphenol-coated GNPs synergistically improve the radical scavenging properties and enhanced the apoptosis of cancer cell.

## Background

Functional nanomaterials are used for various biomedical applications, and it is important to devise a safe and cost-effective alternative approach for targeted cancer therapy. Functionalized gold nanoparticles (GNPs) as an emerging and promising nanocarrier, particularly for the application in nanoparticle-mediated targeted therapy, have shown a significant progress in cancer therapy and diagnosis. Among all nanomaterials, GNPs are more promising due to their unique physicochemical properties, such as biocompatibility, surface plasmon resonance (SPR), and ability to functionalize with amine and thiol groups (Choi et al. 2010; Koshevoy et al. 2011). The therapeutic efficacies of GNPs were demonstrated by various studies which include the multifunctionality and specific interaction of target moieties, such as peptide, antibody, DNA/RNA, and drug with cellular molecular markers (Delong et al. 2010; News and Views in 2010; Thundimadathil 2012). The peptide favors therapeutic application due to its small size, ease of synthesis, tumor-penetrating ability, bioavailability, and good biocompatibility (Agyei and Danquah 2011; Gu et al. 2012; Prades et al. 2012). Peptides such as cell-penetrating peptides, therapeutic peptides, and antioxidant peptides have been reported earlier for cancer therapeutic applications (Thundimadathil 2012; Flora and Pachauri 2010 ; Farkhani et al. 2014). The antioxidant molecules increase hydrogen peroxide levels, scavenge reactive oxygen species (ROS), and induce cancer cells to undergo apoptosis (Turkevich 1951). Thiol-based antioxidant systems are present in mammals to provide antioxidant property and, therefore, antioxidant molecules are important molecules for disease prevention (Valko et al. 2006; Zeng et al. 2015).

The redox imbalance in a cancer cell is correlated with the oncogenic stimulation and important indicator of the disease progression. Therefore, it has considered as a pro-tumorigenic signaling molecule that, involved in the initiation, progression and metastasis of cancers (Maeda 2009; Valko et al. 2007; Storz 2005; Droge 2002; Finkel 2003; Kimura et al. 2005). Hence, modulating the redox state of the cancer cell is an interesting area of study for cancer therapy. Several studies reported the nanocarrier-based targeting of the cancer biomarkers such as, transforming growth factor-beta-1 (TGF-β1), transmembrane receptor P185(HER2) for targeted cancer therapy, to our knowledge, none of these studies utilized to target the ROS using antioxidant peptide (Pep-A) (Prades et al. 2012; Tsai et al. 2013; Kodiha et al. 2015). Hence, it is imperative to develop a suitable biocompatible functionalized nanomaterial for specific and effective targeting the cancer tissue.

In this study, we used RB cancer model for targeting the ROS, by means of the antioxidant peptides (Pep-A) which contain alternative aromatic or sulfur-containing amino acid. The side chains of Pep-A are believed to contribute to strong radical scavenging activities of peptides in the cancer cell. Toward achieving this aim, we design novel multifunctional strategies to attain more specific and effective response. The application of antioxidant molecule alone for targeted therapy has limited its application due to bioavailability and stability in biological environment, limitations of which prompted this research of utilizing nanocarrier-mediated delivery, as it could play a pivotal role of being an effective therapeutic agent. Hence, GNPs-Pep-A composite was synthesized using thiotic acid (TA), an antioxidant linker molecule. Thiotic acid improves the metal-chelating capacity, radical scavenging, and improves the antioxidant effect. The GNPs synthesized by *Vitis vinifera, L is* chosen for the delivery of Pep-A due to several advantages associated with the natural polyphenols used in the synthesis processes: (1) antioxidant and anticancer property, (2) reduced chemical and metal toxicities (3) synergistic improvement in the antioxidant effect of Pep-A, and (4) most importantly, decrease in the nanoparticle-mediated ROS induction (Sharma et al. 2011). The current findings indicate that bioconjugate (GNPs-Pep-A) shows therapeutic efficacy by quenching the ROS species in the cancer cells. The antioxidant potency of bioconjugate to target the ROS is owing to the co-existence of different phases in a conjugate (GNPs-Pep-A).

Although a number of nanocarrier-based approaches have emerged for cancer therapy, this study is an aid for ROS-mediated targeted therapy. It is an effective and safe approach for the diseases like cancer which are highly heterogeneous in nature. Thus, the current study provides a rational plan with an alternative to conventional peptide-functionalized targeted therapy, which would be an effective strategy in the clinical management of cancer.

## Methods

### Evaluation of antioxidant effect of peptides A and B (Pep-A and Pep-B)

Two short peptides, Pep-A and Pep-B, were selected and evaluated for antioxidant efficiency by measuring their effects on cellular reactive oxygen species (ROS) and superoxide dismutase (SOD) enzyme activity on cultured Y79 retinoblastoma cells. The antioxidant peptides, Pep-A: Pro-His-Cys-Lys-Arg-Met (PHCKRM) and Pep-B:Thr-Arg-Asn-Tyr-Tyr-Val-Arg-Ala-Val-Leu (TRNYYVRAVL), were procured from AnaSpec, Bi Biotech India Pvt Ltd, New Delhi. The thiotic acid-modified Pep-A peptides were custom synthesized by Custom Peptide Synthesis, USV limited India, CPS 1514-1. The peptides were HPLC purified to obtain >95 % purity.

#### Cell culture

Y79 retinoblastoma cell line (RCB1645) was obtained from RIKEN BioResource Center cell bank (Ibaraki, Japan) and maintained with an atmosphere containing 5 % CO_2_ at 37 °C in RPMI 1640 media (Sigma-Aldrich, USA) with 10 % FBS. After it attains 80–90 % confluence, the cells were used for all the in vitro experiments. Muller-glial (MIOM1) cell line derived from the neural retina (Limb et al. 2002) was grown in DMEM media supplemented with 10 % FBS and maintained at 37 °C with 10 % CO_2_. Muller-glial cell line was a gift from Dr. G. A. Limb, the Institute of Ophthalmology, University College, London.

#### Measurement of intracellular ROS levels on peptide treatment using 2′, 7′ Dichlorodihydrofluorescein (DCF) assay

The intracellular ROS levels were measured using an OxiSelect ROS Assay Kit (Cell Biolabs Inc, CA, USA) as per the manufacturer’s instructions. Y79 cels, RB cell lines, were seeded at 7 × 10^3^ cells/well in 96-well plates and incubated at 37 °C overnight. Then, the cells were washed thrice with sterile PBS and treated with 100 μl of 1X DCFH-DA (2′, 7′-Dichlorodihydrofluorescin diacetate) for 1 h at 37 °C. The solution was removed, and the cells were washed with sterile PBS and treated with varying concentrations (ranging from 10 to 100 µM) of Pep-A and B for 24 h. The reaction was terminated by adding 100 μl of cell lysis buffer, mixed thoroughly for proper cell lysis, and incubated for 5 min. The fluorescence was read on a Spectra Max M4 multi-detection micro plate reader (Molecular devices, CA, USA) at 480-nm excitation/530-nm emission. The ROS levels in cell lysates were then calculated using the DCF standard curve.

#### SOD activity measurement

Y79 cells were seeded at 2 × 10^5^ cells/well in 12-well plates with 1000 μl of culture media and incubated at 37 °C overnight. The cells were then exposed to varying dosages of Pep-A and Pep-B (10, 50, and 100 μM) in fresh medium and then incubated for 6 h. At the end of the incubation, the cells were collected and washed with ice-cold PBS twice and lysed with cell lysis buffer [0.1 M Tris/HCl, pH 7.4 containing 0.5 % Triton X-100, 5 mM β-Mercaptoethanol, 0.1 mg/ml PMSF]. Cell lysate was centrifuged at 14000×*g* for 5 min at 4 °C. The supernatant contains a combined SOD activity from cytosol and mitochondria. The SOD activity was measured using the superoxide dismutase (SOD) activity assay kit, (BioVision, CA, USA, Sigma Aldrich, MO, USA) as per the manufacturer’s instructions. In brief, the cell lysate, buffer, enzyme, and the WST reagent were diluted, and the solution was added as per the protocol and incubated at 37 °C for 20 min and read at 450 nm on a microplate reader (Biotek, VT, USA). SOD activity was then calculated as a percentage of the inhibition activity of xanthine oxidase.

#### Cytotoxicity assay

Y79 retinoblastoma and MIOM1 cells were seeded at 5 × 10^3^ cells/well in 96-well plates and incubated at 37 °C overnight. The cells were then treated with varying concentrations (10, 30, 60, and 100 µM) of Pep-A and Pep-B in a fresh medium and incubated for specific time periods (24 and 48 h). At the end of the incubation, 10 µl of MTT [3-(4, 5-dimethylthiazol-2-yl)-2, 5-diphenyltetrazolium bromide; 5 mg/ml] was added to the cells with fresh medium (100 µl) and incubated at 37 °C until formazan crystals were formed. The formazan crystals were dissolved in 100 µl of DMSO, and the reading was taken at 570 nm (BioTek, ELISA, USA). Cell viability was calculated as Test OD/Control OD × 100.

### Preparation of bioconjugate (GNPs-Pep-A)

The filtered GNPs were used to prepare the bioconjugate (GNPs-Pep-A), and the conjugation protocol was followed as described by N. Chanda et al (Chanda et al. (2010). The thiotic acid (TA)-modified peptide conjugates with GNPs in different ratios of peptides, for example, GNPs:Pep-A_1_ [(18 ml GNPs: 1.7 mg) and GNPs:Pep-A_2_ (18 ml GNPs: 4.3 mg)] were used. The conjugates were prepared using 18 ml of GNPs (OD_520_ nm = 1.00), [2.32 × 10^−9,^ Molar particles/L, (242 × 10^−6,^ Moles of gold/L)] mixed with 4.3 mg of Pep-A dissolved in 1 ml of water and kept under stirring overnight. A similar protocol was followed for the preparation of other conjugate, and the synthesized conjugate was subjected to purification. GNPs-Pep-A was washed twice by centrifugation at 15,000 rpm for 30 min with distilled water to remove unattached peptides on the surface of the gold. The thiotic acid (TA) has sulfur-containing antioxidant linker molecules that bind with GNPs to form Au–S bond between peptide and GNPs (Yin 2007). The GNPs-Pep-A was pelleted, and the supernatant was measured for the presence of peptide by reading the absorption at 280 nm. The 50 µM was the final concentration of GNPs-Pep-A which was used for the in vitro studies. The conjugate (GNPs-Pep-A) concentration was measured using the GNP’s concentration.

### In vitro stability of GNPs-Pep-A

The stability of GNPs-Pep-A was studied in a variety of solvents and buffers using previously reported methodology (Kalmodia et al. 2013). One ml of GNPs-Pep-A was mixed with 0.5 ml (10 mM) each of DTT, cysteine, histidine, and NaCl; also, GNPs alone was used as a reference. The solutions were then incubated for 2 h at room temperature, and the stability of the GNPs-Pep-A was determined using UV–Vis spectrophotometer (DU 800 Spectrophotometer, Beckman Coulter Inc, CA, USA).

### UV–Vis spectroscopy of GNPs-Pep-A

The GNPs-Pep-A were characterized using UV–Visible spectroscopy (Molecular device-M4, CA, USA) to observe the surface plasmon resonance (SPR) with respect to the GNPs. The spectra were obtained in the range of 400–800 nm using water as a blank.

### FT-IR characterization of GNPs-Pep-A

The lyophilized GNPs-Pep-A was subjected to Fourier transform-infrared spectrum (FT–IR) analysis for characterizing the functional groups using an FT–IR Spectrometer (Vortex 70 BRUKER, Billerica, MA 01821, USA). The samples were mixed with KBr powder, pulverized, and formed into a disk-shaped pellet. FTIR spectra were recorded in the frequency range of 500–4000 cm^−1^ in transmittance mode.

### Size and zeta potential measurement by dynamics light scattering (DLS) of GNPs-Pep-A

The average hydrodynamic size (*Z* average), size distribution (% number distribution), and zeta potential of GNPs-Pep-A as a colloidal suspension were measured using dynamic light scattering (DLS) and a Zetasizer [Nano-ZS model equipped with 4.0 mW, 633 nm laser, Model ZEN3600, serial no. MAL1024433, Malvern Instruments Ltd., Malvern, UK]. The particle size was determined based on the Brownian motion relating to the size of the particles suspended in a liquid.

### Uptake and internalization by fluorescent-activated cell sorter (FACS)

The Y79 cells were seeded on the lysine (0.01 mg/ml)-coated glass slide. The cells were cultured overnight and treated with different concentrations (50 or 100 µM) of FITC-tagged Pep-A for 4 h, for studying the localization and internalization of the peptide by fluorescence microscopy. The FITC-labeled peptide A molecules (molecular mass 771.2) were custom synthesized (CPS 1468-1, custom peptide synthesis, USV limited India) with ~95 % purity obtained by HPLC method. The GNPs and GNPs-Pep-A conjugates were doped with the R6G dye as described by Kalmodia et al., for the internalization study (Kalmodia et al. 2013). The internalization of the peptide, R6G-doped GNPs and GNPs-Pep-A after 4 h was qualitatively detected by Zeiss LSM, 710 laser microscope supplied by Axio vision (Germany). The peptide uptake was performed by flow cytometric analysis to confirm the concentration-dependent uptake of the peptide in cell culture in vitro. Y79 cells were seeded at 2 × 10^5^ cells/well in six-well plates and treated with 50 and 100 µM concentrations of Pep-A for 4 h. The cells were then spun at 1500 rpm for 5 min, washed with PBS buffer, and then resuspended in FACS buffer. The cells were then analyzed for uptake of the peptide using FACS Calibur (BD Biosciences, CA, USA) by means of CELL QUEST PRO software. The fluorescence of FITC-stained cells was excited with an argon laser at 488 nm, and the emissions were detected at 530 nm. The overlay graph and scattered plot were used to confirm the FITC positive cell population.

### Functional effects of GNPs-Pep-A

Cytotoxic effects of GNPs and Pep-A were evaluated by MTT assay as per the protocol described in “Cytotoxicity assay” section. Antioxidant effects of GNPs-Pep-A, bioconjugate were evaluated by measuring the cellular ROS and SOD enzyme activity by similar methods as described in “Measurement of intracellular ROS levels on peptide treatment using 2′, 7′ dichlorodihydrofluorescein (DCF) assay” and “SOD activity measurement” **sections**, respectively. In these assays, the peptides were replaced with the GNPs and GNPs-Pep-A. For evaluating the effects of GNPs-Pep-A on mRNA expressions of antioxidant genes—superoxide dismutase (SOD), glutathione peroxidase (GPX), and catalase (CAT)—retinoblastoma cells (Y79) were seeded at a density of 1 × 10^6^ cells/well, and treated with GNPs and GNPs-Pep-A for 24 h. Total RNA was isolated using Trizol, and concentration of RNA was quantified using a Nanodrop (Biospec-Nano,Shimadzu, Biotech, Japan). The total RNA was converted into cDNA using an Applied Biosystem kit (High-Capacity cDNA Transcription Kit, USA). Quantification of target gene expression was performed in triplicate in a 20-µl reaction mixture containing 0.1 µg of cDNA, specific primers (Table 1), and SYBR green reagent in 96-well plates on a real-time PCR system (Applied Biosystems, Singapore). GAPDH and β actin were used as internal controls, and the detection was carried out by measuring the binding of fluorescence dye SYBR green to double-stranded DNA. After cycling, relative quantitation of the tested gene cDNA against the internal control was performed using the ΔCT method. The relative amount of gene-specific mRNA to housekeeping gene was calculated using 2^−ΔΔCt^, where Ct, the number of cycles at which amplification reaches a threshold, is determined using SDS software version 1.3.

**Table 1:** Primer sequences for target genes used in qRT-PCR

Gene	Primer sequence	Product size
Catalase (CAT)	FP 5′- TCTGGAGAAGTGCGGAGATT-3′ RP 5′- AGTCAGGGTGGACCTCAGTG-3′	190 bp
Superoxide dismutase 1 (SOD1)	FP 5′- GATGAAGAGAGGCATGTTGGAGAC-3′ RP 5′- GTCTTTGTACTTTCTTCATTTCCACC-3′	186 bp
Glutathione peroxidase (GPX)	FP 5′- GCACCCTCTCTTCGCCTTC-3′ RP 5′- TCAGGCTCGATGTCAATGGTC-3′	207 bp
Glyceraldehyde-3-phosphate dehydrogenase (GAPDH)	FP 5′-GCCAAGGTCATCCATGACAAC -3′ RP 5′-GTCCACCACCCTGTTGCTGTA-3′	498 bp

### Statistical analysis

The in vitro data were analyzed by one-way ANOVA using SPSS-17 software, and the post hoc test was performed for multiple comparisons. Student’s unpaired *t* test was used for statistical comparisons, and *P* < 0.05 was considered significant.

## Results and discussion

**Fig. 1:**
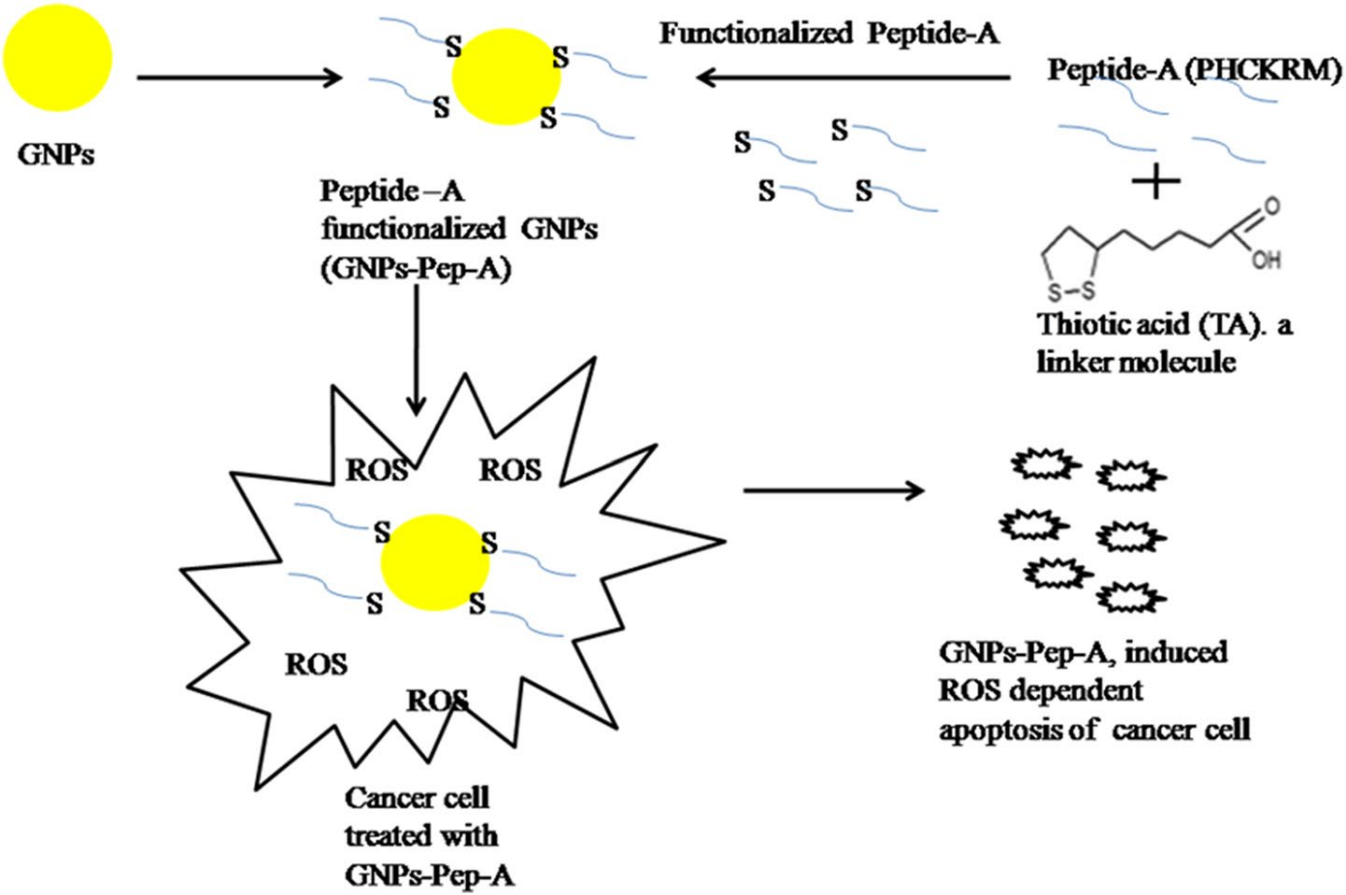
Schematic of GNPs -Pep-A synthesis and antioxidant effect. GNPs-Pep-A synthesis. Schematic presentation of chemical reaction for functionalization of GNPs with thiotic acid-modified Peptide-A and its effect in cancer cell in vitro

The results obtained in the current study highlights the significance of bioconjugates on free radical scavenging property. The peptide-functionalized GNPs reduce the metal ion toxicity owing to peptide coating on the GNPs surface. The bioconjugate provides the stability to peptide and also imparts new functionalities in the synthesized GNPs-Pep-A. It shows the large synergistic effect on ROS reduction due to the coexistence of the two phases in close proximity, although the chemical reactivity of individual phase is significantly different from the other (Sanchez et al. 2011). It has been reported that the organic–inorganic conjugate material retains its original property and helps to maintain its stability, thermal behavior, aqueous solubility, and shows novel properties (Sanchez et al. 2005). Figure 1, a schematic illustration, illustrates preparation of GNPs-Pep-A conjugate and its radical scavenger capability in cancer cell.

### In vitro antioxidant activities of peptide A and peptide B

**Fig. 2:**
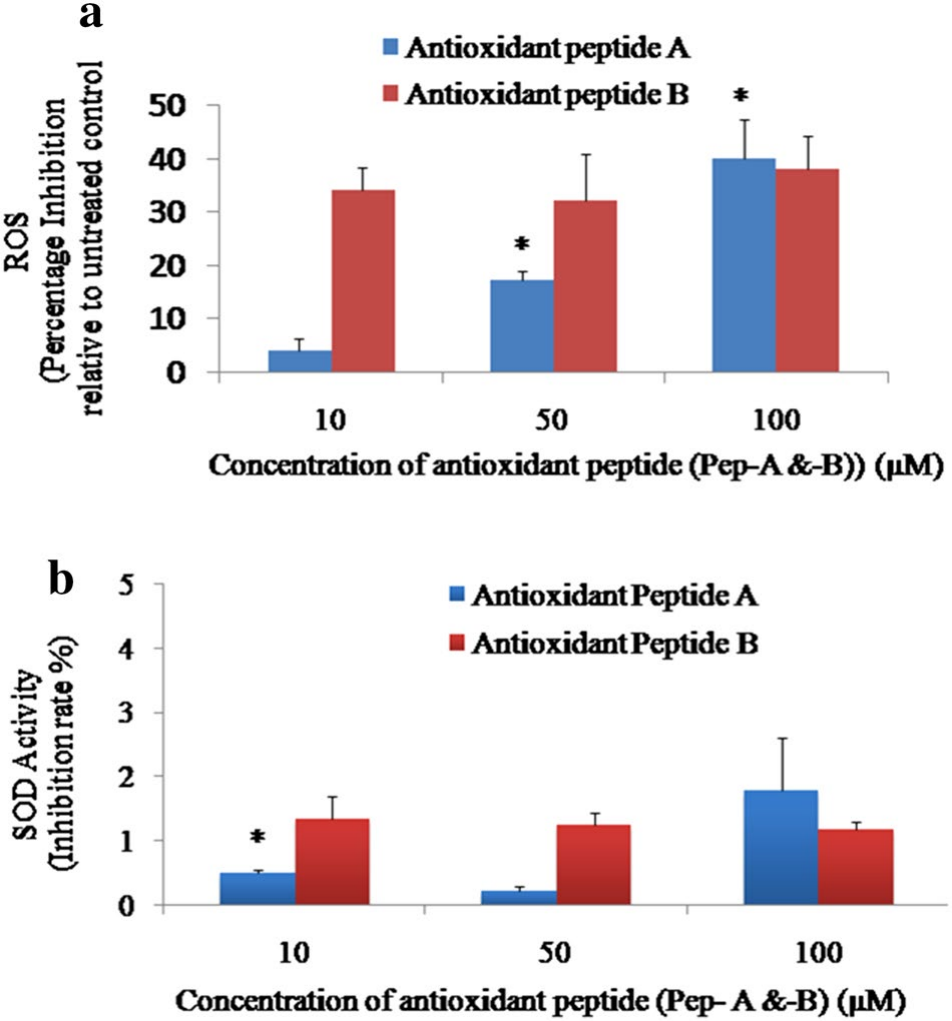
Evaluation of antioxidant activities of Pep-A and Pep-B, inhibitions of ROS and SOD levels by antioxidant peptide A. **a** Antioxidant activities of Pep-A and Pep-B were evident from the decrease in ROS levels in Y79 RB cells on peptide treatment. The percentage decrease in ROS levels relative to untreated control can be noticed. * indicates the significant difference at *P* < 0.05 relative to 10 µM of peptide A. **b** Antioxidant Pep-A- and Pep-B-treated Y79 RB cells showed initial decrease in SOD activity when treated at 10–50 µM, and at 100 µM, the enzyme activity increased. SOD activity was calculated as a percentage of the inhibition activity of xanthine oxidase and depicted as increase/decrease relative to untreated control. *Data points* indicate mean ± SEM of duplicate values. * indicates the significant difference at *P* < 0.05 relative to 50 µM of Pep-A

The antioxidant activities of two peptides were determined by measuring the intracellular ROS levels and the cellular principal antioxidant enzyme, and superoxide dismutase (SOD) activity. Reactive oxygen species (ROS) are formed as a by-product during normal cellular metabolism and also during physical and environmental stress to the cell. These ROS cause damage to DNA, oxidize amino acids and lipids, and inactivate important enzymes. Cells scavenge these species with the help of first-line defense enzymes: Glutathione peroxidase (GPX), catalase (CAT), and superoxide dismutase (SOD). Therefore, ROS levels and the key antioxidant members were estimated for peptide treatments to understand their roles in the regulation of cellular oxygen stress. Pep-A showed a dose-dependent inhibition of ROS levels ranging from 4 to 40 % relative to untreated control. In contrast, Pep-B showed inhibition in the range of 32–38 %, and the inhibition levels were inconsistent (Fig. 2a). Methionine and cysteine in our peptides may donate sulfur and hydrogen making them effective free radical scavengers compared to Pep-B. In agreement with the observed results, in earlier studies also, proteins and peptides with sulfur-containing amino acids and aromatic side chains have been reported as effective regulators of the oxidative damage (Elias et al. 2008).

The effects of 10–100 μM of Pep-A concentrations were studied on the SOD enzyme activity. The enzyme activity decreased by 0.5 and 0.7-folds at 10 and 50 µM Pep-A concentrations, respectively (Fig. 2b), and increased by 1.79-folds at 100 µM Pep-A treatment, indicating that this concentration could be ideal for the treatment on Y79 a, RB cells (Fig. 2b). Furthermore, the Pep-A could be involved in decreasing the ROS (Fig. 2a) by increasing the antioxidant enzyme activity. A similar increase in the antioxidative enzyme levels in the presence of Hoki skin antioxidative peptide in hepatocarcinoma cells is attributed to the peptide’s role in maintaining the redox balance in the cellular environment (Jridi et al. 2014). The concentration- and time-dependent effects of inhibition of Lypooxigenase 12 enzyme levels by a peptide were reported in the breast cancer cells. This study suggested that the therapeutic effect of the peptide depends on concentration, cell line, and the duration of treatment (Singh et al. 2012). Pep-B used in the study did not show any increased SOD enzyme activity at different concentrations (Fig. 2b), indicating that this peptide did not elicit any change in the cellular antioxidant enzymes. Therefore, further work is carried out using Pep-A. The hybrid bio-nanoparticle is synthesized using GNPs, 100 µM Pep-A, and thiotic acid as linker molecules. The concentration of the peptide used was 100 µM which was found to be effective to elicit the antioxidant enzyme response by this peptide (Fig. 2b).

### Characterization of bioconjugate (GNP-Pep-A)

**Fig. 3:**
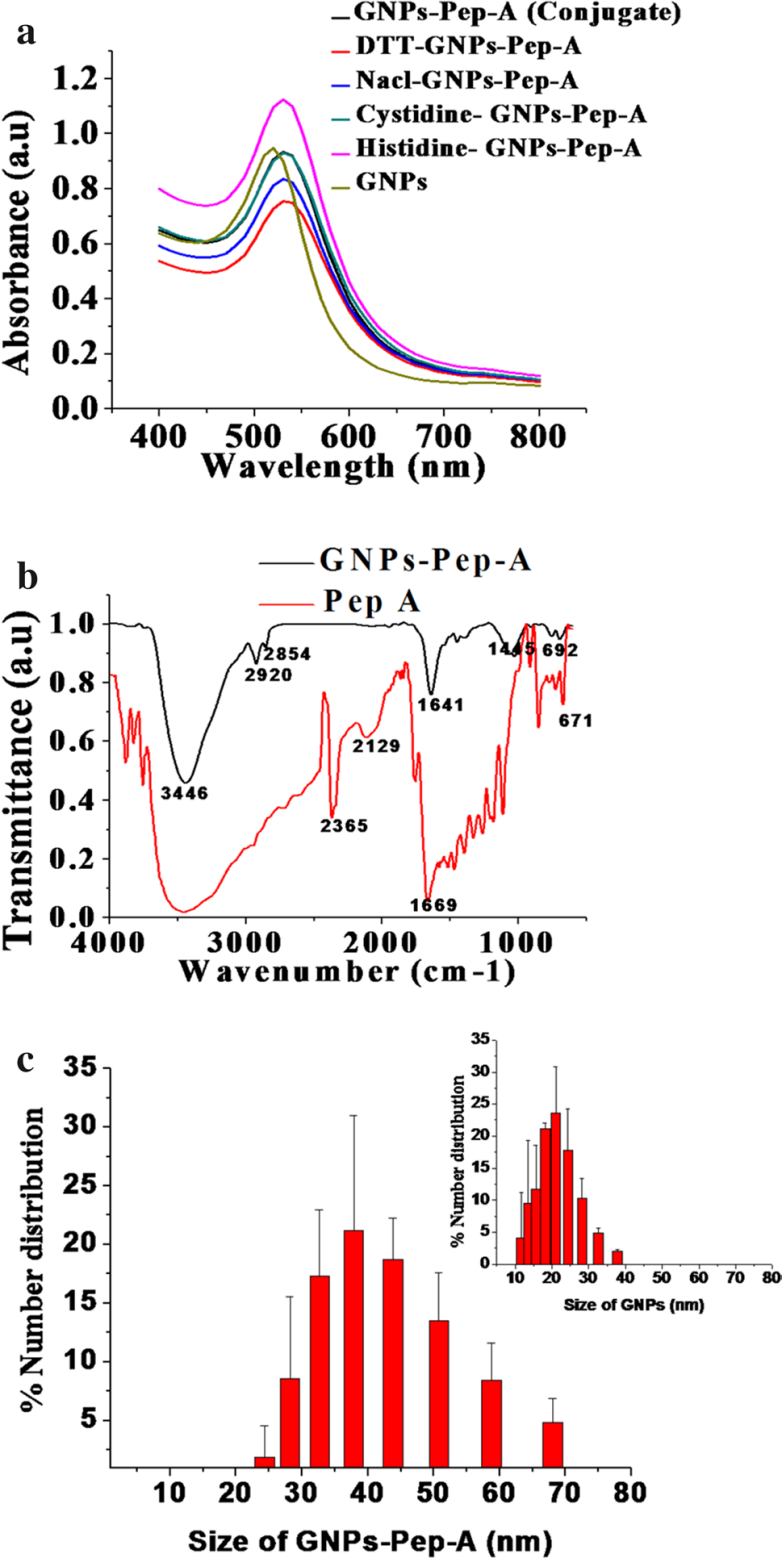
Characterization of GNPs-Pep-A by UV–visible spectra of GNPs and conjugate. **a** The *graph* shows a shift in SPR for GNPs-Pep-A (*black* which is actually not visible but is merged with *green line*), and for GNPs-Pep-A in different buffers, no alteration in the shift confirms the stability of the conjugate in vitro; **b** size distribution of GNPs-Pep-A (Inset: GNPs); **c** the FTIR spectral difference and the functional group confirming GNPs-Pep-A conjugate formation

The presence of the peptide on the GNPs was studied using DLS, FTIR, and UV–Visible spectroscopy (Fig. 3 a and c). The surface plasmon absorbance band peak of GNPs shows a Red shift at 540 nm after the addition of Pep-A (Fig. 3a). The similar red shift in the surface plasmon absorbance peak was observed after the binding of cysteine molecules to the GNPs. The red shift in plasmon absorbance is attributed to the presence of peptides on GNPs (Maus et al. 2010).

The Pep-A bind to GNPs, which is evaluated by means of FTIR vibrational spectroscopy as this method gives specific vibrational patterns for the ligand bound to GNPs (Tiwari et al. 2014). Spectroscopic analysis of GNPs-Pep-A by FTIR (Fig. 3c) showed vibrational bands at 3446–2365, 2620, and 1641–663 cm^−1^. The presence of 1641 and 1669 cm^−1^ bands in the FTIR spectra of Pep-A and GNPs-Pep-A confirms the presence of the amide bonds. The peptide coating on the GNPs exhibited a characteristic IR band at 3448 cm^−1^, indicating the presence of O–H and COOH moieties, whereas 2920 and 2854 cm^−1^ bands can be assigned to the symmetric and anti-symmetric vibration bands for CH_2_ and CH_3_ groups and C = O stretching of an amide I. In a previous study, COO− bands assigned at 1600 and 1390 cm^−1^are asymmetric and symmetric, whereas a band at a broad range 3000–3500 cm^−1^ range was assigned for NH_3_^+^of amino acid (Khlebtsov 2011). The GNPs-Pep-A nanoparticles showed significant peak shift from 1641 to 1669 cm^−1^ compared to Pep-A alone indicating the effect of metal surface on selective surface–induced functional group shift. Similar changes in vibrational modes in the presence of metallic nanoparticles have been observed before (Alexander et al. 2002). The shift in the vibration peak intensities may indicate covalent binding of Pep-A to the GNPs to form GNPs-Pep-A (Aryal et al. 2006). The peaks at 2129 and 2365 cm^−1^ in the spectra of the Pep-A indicate S–H bond in the peptide molecule, whereas the disappearance of these peaks in GNPs-Pep-A may suggest the absence of S–H bonds and the presence of the Au–S bond (Kalmodia et al. 2013).

### Stability of the GNPs-Pep-A

**Fig. 4:**
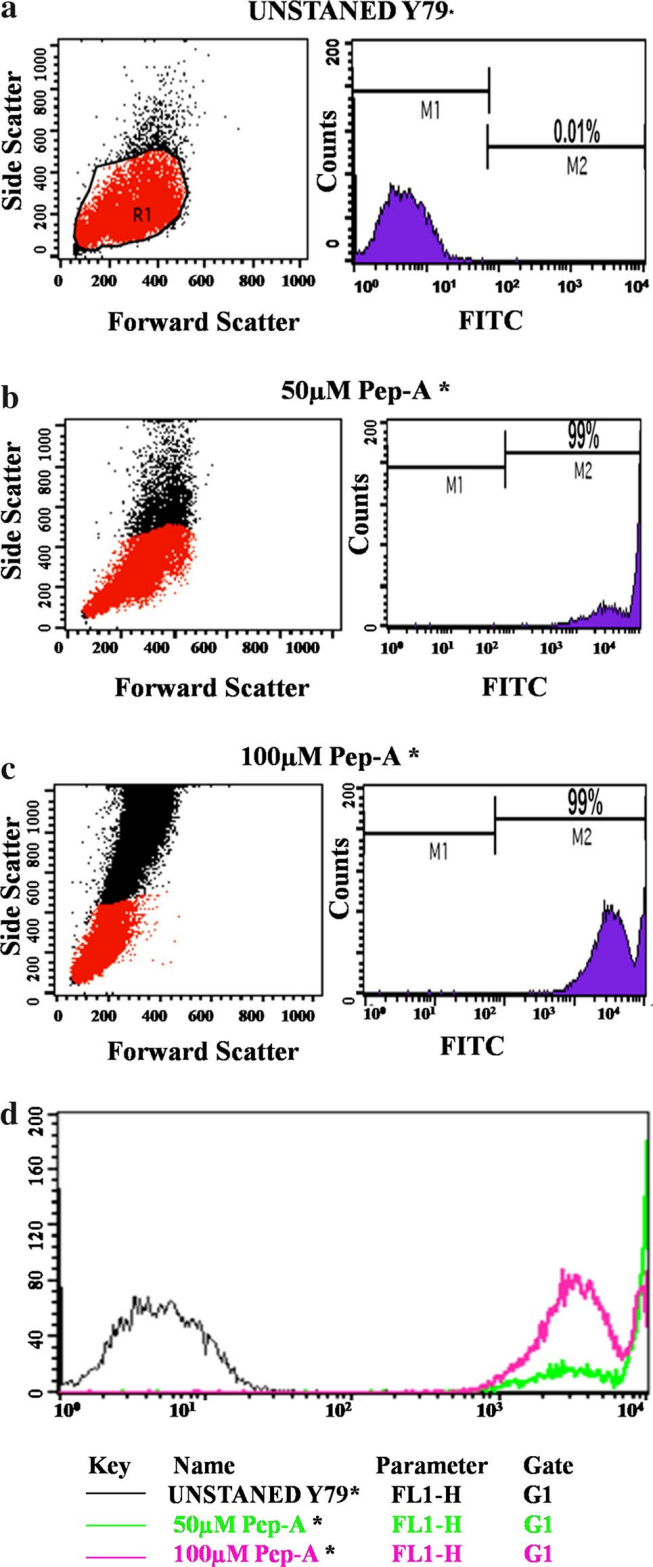
Cell uptakes of antioxidant peptide A in Y79 RB cells evaluated by flow cytometry. **a** Untreated Y79 RB cells, **b** and **c** cells treated with 50 and 100 µM of Pep-A showing maximum (99 %) number of FITC positive population in M2. **d** Overlay picture showing peptide uptakes in control and treated Y79 RB cells

The number and size distribution of GNPs-Pep-A particles were measured by DLS. The size of the nanoparticles was in the range of 10–22 nm (Fig. 3a and 4b), whereas the zeta potential of GNPs was in the range of −17 to −21 mV (Table 2). The high negative potential indicates that the nanoparticles are stable. The hydrodynamic size of the GNPs is 49.6 ± 0.4 nm, and the size increased to 91.7 ± 1.7 in GNPs-Pep-A nanoparticles. The increase in hydrodynamic size could be due to the presence of peptide molecules capped on the surface of the GNPs (Table 2). The DLS measurements (Fig. 3a) indicate a monodispersion of GNPs-Pep-A in the solvent water (Aryal et al. 2006). The increase in hydrodynamic size of the GNPs after conjugation to bombesin peptide has been reported earlier (Chanda et al. 2010). The increasing bombesin peptide concentration on the GNPs increased the hydrodynamic size of the nanoparticles (Au–BBN-1115, Au–BBN-2137, and Au–BBN-2, 155), whereas the core size of nanoparticles was found to be16 ± 7 nm. Similarly, the zeta potential of the GNPs-Pep-A (−26.5 ± 0.2) is larger than that of GNPs alone (−22.5 ± 0.2 mV), indicating a greater stability of these nanoparticles than the GNPs in vitro. (Table 2).

**Table 2:** Effects of different solvents on stability of GNPs-Pep-A

Sample	Zeta potential (mV)	Z Average (hydrodynamic size)
GNPs	−22.5 ± 1.77	49.6 ± 0.4
Conjugate (GNPs-Peptide A)	−26.5 ± 2.02	91.17 ± 1.17
DTT + conjugate	−35.4 ± 3.95	92.36 ± 0.30
NaCl + conjugate	−22.6 ± 1.98	89.48 ± 0.31
Cysteine + conjugate	−	91.05 ± 0.99
Histidine + conjugate	−32.0 ± 0.25	95.96 ± 0.88

The stability of the GNPs-Pep-A was tested in different solvents using UV–Visible spectroscopy and DLS measurements (Fig. 3a and b). The absorption peak of GNPs-Pep-A at 540 nm remained unaltered in various solvents except for the solvent-containing histidine which shows increase in absorbance (Fig. 3a) indicating the binding activity to form GNPs-Pep-A hybrid. The histidine has a positive charge on its imidazole functional group which brings about changes in the binding affinity with the metal nanoparticles (Flora and Pachauri 2010). The zeta potential of GNPs-Pep-A-His (32.0 ± 0.25) is higher than GNP-Pep-A(−32.0 ± 0.25) indicating a greater stability in the presence of histidine (Table 2). The sulfur-containing amino acid cysteine and DTT also increased the zeta potential to −36.96 ± 1.17 and −35.4 ± 3.95, respectively (Table 2). The cystine amino acid could increase the acidity of the solution thereby increasing the zeta potential of the nano particle as evident from the increased potential in the presence of DTT and amino acid. In the presence of high ionic strength salt NaCl, the zeta potential does not alter. The conjugated Pep-A to GNPs maintains the electric double layer of the GNPs and decreases the probability of the salt Na^+^ ions to cause any aggregation to the hybrid (Bei et al. 2011; Liu et al. 2007). Thus, the GNPs-Pep-A is stable in various biologically relevant solutions, making these characteristics suitable for their potential applications.

### Internalization and uptakes of Pep-A, GNPs-Pep-A, and GNPs

**Fig. 5:**
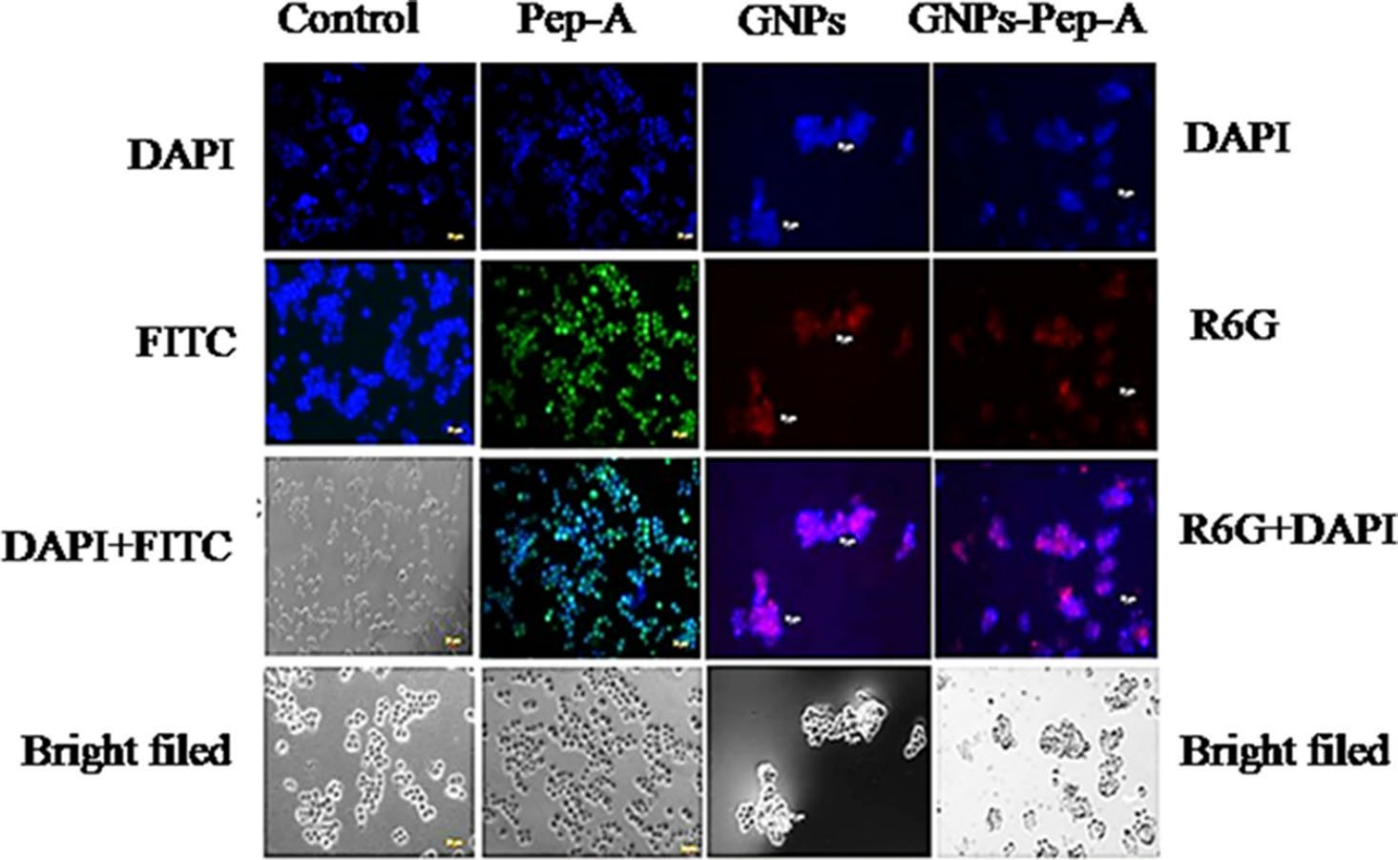
Cell uptake of antioxidant peptide A in Y79 RB cells evaluated by fluorescence microscopy. Micrographs showing internalization of FITC-labeled peptide Pep-A after 3 h of treatment in Y79 RB cells. panel-1: control, panel-2: Pep-A, panel-3: GNPs, and panel-4: GNPs-Pep-A. Magnification 10 × Magnification: 10× (*Scale bar*: 10 μm)

The Peptide-A (Pep-A) uptake was measured by the FACS (fluorescence-activated cell sorter) and microscope using Y79 cells, RB cell line (Figs. 4, 5). The FACS analysis results for Pep-A showed a concentration-dependent uptake into the Y79 cells. The histogram statistic indicates that there is a significant shift in the FITC positive population (Fig. 4 a, *X*-axis) from M1 to M2. In the control samples (cells without Pep-A), M1 population is 99.99 whereas the M2 population is 0.01; in the case of 50 µM of Pep-A, M1 population is 99.97, whereas the M2 population is 0.03. Similarly, in 50 µM of Pep-A, M1 population is 99.95, and the M2 population is 0.05. The shift of the population from M1 to M2 in the peptide is an indication of the peptide internalization. In addition to the histogram statistic, overlay graph (Fig. 4 b) further confirms the concentration-dependent internalization of the peptide into the Y79 RB (Fig. 4b). The increase in the number of counts (Y axis, Fig. 4 b), as well as the FITC positivity M2 population compared to the control, indicates that peptide is internalized by the Y79 cells (Hällbrink et al. 2004). It has been reported that the uptake of the peptide is mainly by CPP (cell-penetrating peptide) that follows the endocytotic pathways, endocytosis, macropinocytosis, and clathrin-mediated endocytosis. The proteoglycan contributes mostly to facilitate internalization of the peptide by increasing the concentration of the peptide at the membrane surface by enhancing the recruitment of peptide via electrostatic interactions and helps in reorganization of the F-actin to facilitate the internalization of the peptide (Kosuge et al. 2008). The amino acid composition also significantly alters the internalization of the peptide (Amand et al. 2012). Arginine is an important amino acid, which enhances the internalization of the peptide;this could be due to the cationic nature of arginine, which disrupts the membranes of macropinosomes, and this may lead the peptides into the cytosol. Alternatively, hydrophobic counter-anion molecules may help the translocation of arginine peptides into the cytosol. In the case of Pep-A (PHCKRM), the P (Proline) and M (Methionine) amino acids are hydrophobic in nature which could help in internalization of peptide in addition to the arginine (R) (Guterstam et al. 2009). The internalization of the nanoparticles is further visualized by the presence of red fluorescent signal from the cells treated with the R6G dye-doped GNPs and GNP-Pep-A nanoparticles (Fig. 5 panel 3 and 4). The R6G-doped GNPs have been successfully detected by fluorescence due to presence of dye on the surface of metals (Chen et al. 2007). In our current results, the GNPs (Fig. 4 panel 3) and GNPs-Pep-A (Fig. 5 panel 4) show red fluorescence of R6G dye on GNPs. However, when FITC-conjugated peptide was attached to GNPs, green fluorescence from the dye was not observed (Fig. 5 panel 4). This could be due to the fluorescence quenching by the proximity of the metal atoms in GNPs (Oishi et al. 2009).

The optical microscopic images of the Y79 cells, RB cell line, treated with the peptide, GNPs, and GNPs-Pep-A (Fig. 5) show the morphological differences compared to the untreated cells. The cells treated with the peptide alone (Fig. 5 panel-2) show similarity in morphology as the control untreated cells (Fig. 5 panal-1), whereas the cellular morphologies of treated cells with GNPs and GNPs-Pep-A (Fig. 5 panel 3 and 4) showed significant differences. The cell fragments were observed, indicating apoptotic bodies formed during the process of membrane cell death. The clustering of cells, shrinkage, change in size, and condensation of cytoplasm is in accordance with the apoptotic or necrotic cell death and are the most commonly observed microscopic features (Barros et al. 2003). The better performance of the GNPs and GNP-Pep-A compared to the peptide alone in apoptosis of cancer cells indicates the potential application of nanoparticles in cancer therapy.

### Antioxidant function of GNPs and GNPs-Pep-A

**Fig. 6:**
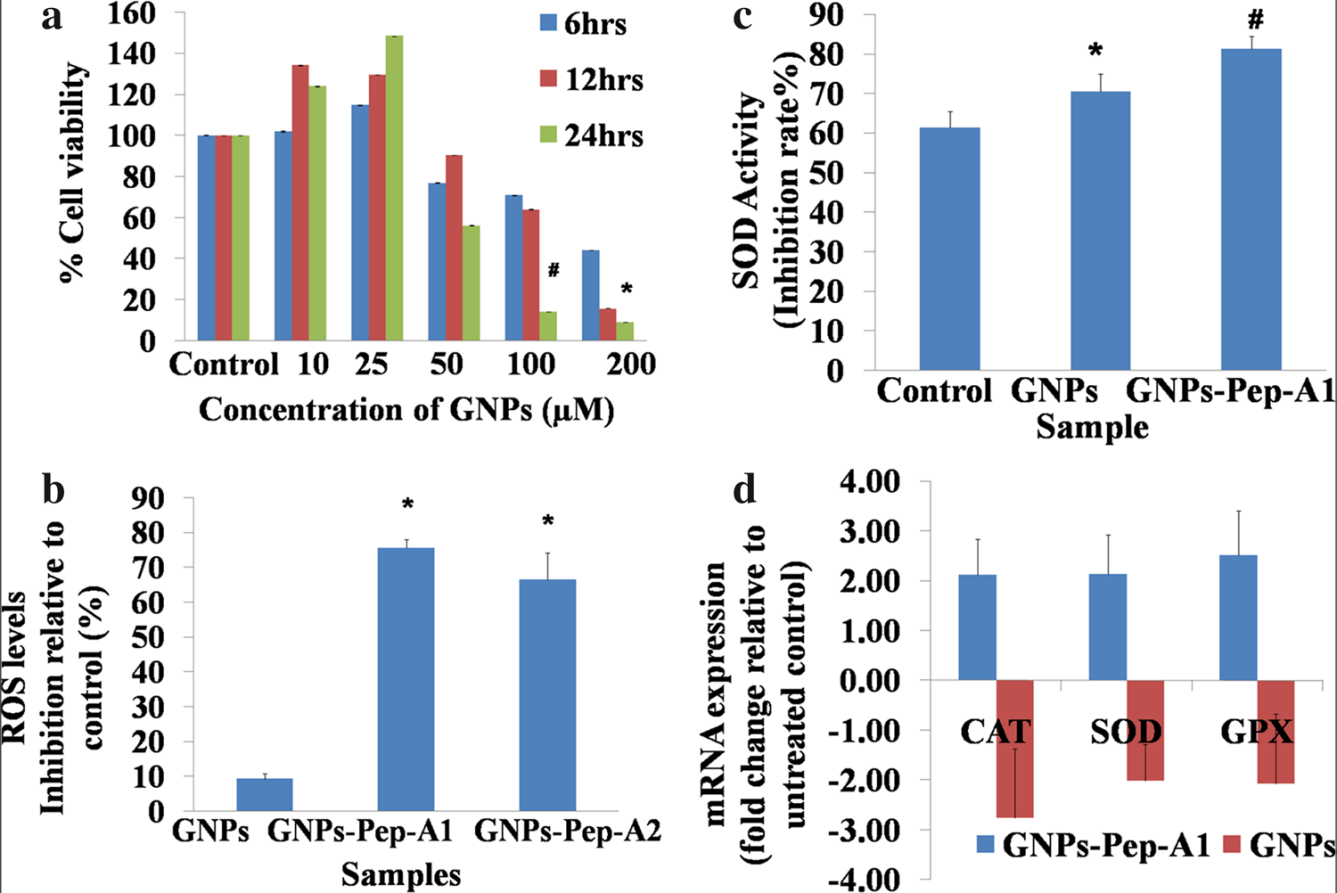
Functional studies with GNPs and GNPs-Pep-A bio conjugate. **a** Native GNPs treated on Y79 RB cells at varying dosages for 6, 12, and 24 h showed both dose- and time-dependent decreases in cell viability. # indicates statistical significant difference with respect to the controls for 50 µM at 24 h, whereas # indicates significant differences at 6, 12, and 24 h for 100 µM with respect to control at *P* < 0.05. **b** GNPs-Pep-A decreased the ROS levels in Y79 RB cells effectively compared to GNPs alone and peptide alone (Fig. 2a). The final concentration of 50 µM GNPs (Moles of gold) used for functional study. GNPs-PepA_1_ and GNPs-PepA_2_ contain the final concentrations of Pep-A being 100 and 250 µM, respectively. Each column indicates mean percentage inhibition of ROS levels from triplicate values relative to untreated control. *Error bars* SEM from triplicate values. * indicates the significant difference relative to GNPs at *P* < 0.05. **c** GNPs-Pep-A increased the SOD enzyme activity by 1.3-fold compared to 1.1-fold increase by GNPs alone. Each column indicates SOD activity (inhibition rate  %) from triplicate values. *Error bars* indicate SEM from triplicate values. * Represents the statistical significant difference with respect to control at *P* < 0.05; # represent the significant difference relative to GNPs at *P* < 0.005. **d** Y79 RB cells treated with GNPs and GNPs-Pep-A were analyzed for mRNA expression of first-line defense antioxidant enzymes. GNPs and GNPs-Pep-A downregulated and upregulated the *SOD* (superoxide dismutase), *GPX* (glutathione peroxidase), and *CAT* (catalase) enzymes’ gene expression levels, respectively. Each column indicates mean ± SEM. of duplicate values

The functional activities of the GNPs and GNPs-Pep-A were studied after establishing the uptake and internalization of these particles into the Y79 cells. The GNPs-Pep-A shows the cooperative effect due to the different functional moieties present in the structure of the conjugate. Therefore, ROS levels and the key antioxidant members were estimated for GNPs and GNPs-Pep-A treatments to understand their roles in the regulation of cellular oxygen stress. The effective concentration of Pep-A for scavenging ROS was found to be 100 µM (Fig. 2), and hence, the ROS levels were measured at this peptide concentration. ROS levels decreased significantly (75 % at *P* < 0.05) with GNPs-Pep-A compared to the GNPs alone (9 %) (Fig. 6a). There was a 66 % decrease in ROS species at 250 µM peptide concentration. Very high dosages of antioxidants may prevent them from exerting their antioxidant action, and may also alter the biological redox state. Combinational therapy with multiple free radical scavengers and sustained release formulations is suggested for an efficient antioxidant therapy (Kohen 2002). Therefore, 100 µM of antioxidant peptide could be the optimum dosage, which showed (Fig. 6a) about 30–40 % decrease in the ROS production compared to other concentrations. The GNPs-Pep-A nanoparticles were more efficient in scavenging ROS than their constituents (GNPs and Pep-A) (Figs. 6b and 2a), indicating a synergistic antioxidant effect due to the combined presence of metal and organic phases in the bioconjugate. It has been reported that the organic–inorganic hybrid material retains its original property along with the improved properties such as stability, thermal behavior, and aqueous solubility, and adds novel properties to the hybrid materials (Sanchez et al. 2005). In the present study, the GNPs-Pep-A hybrid material exhibited better stability and antioxidant capacity compared to its individual constituents.

Further studies were performed to understand the reduction in ROS and its effect on the antioxidant enzyme gene expression. Quantitative real-time PCR analysis with GNPs treatment for the first-line defense antioxidant enzyme gene expression showed twofold downregulations of SOD and GPX, and 2.7-fold downregulation of CAT enzyme mRNA levels (Fig. 6d). GNPs-Pep-A showed 2.1-fold upregulation of the CAT and SOD, and 2.5-fold upregulation of GPX enzyme gene expression (Fig. 6d). The high up-regulated gene expression level of antioxidant enzymes by GNPs-Pep-A compared to GNPs alone signifies that the GNPs-Pep-A could be a stronger antioxidant response regulator. Furthermore, the polyphenols from grapes, thiotic acid as linker molecules are synergistically acting with Pep-A to decrease the oxidative stress. We further tested enzyme activities to confirm the effect of the gene expression. We chose estimation of SOD enzyme activities after the GNP and GNP-Pep-A treatments to study the inhibition activity for GNPs-Pep-A which was 81 % compared to 61 % for the untreated control; GNPs treatment on Y79 RB cells showed 70 % reduction in SOD activity compared to the control (Fig. 6c). The enzyme activities are consistent with the measured SOD gene expression levels. The treatment of GNP-Pep-A resulted in 81 % decrease in the SOD enzyme activities (Fig. 6c) indicating that the compounds present in the nanoparticles are acting as efficient free radical scavengers bringing down the need for SOD enzyme to scavenge them. The enhanced antioxidant capacity of GNPs-Pep-A could be attributed to the presence of polyphenols on the GNPs, TA, and the presence of antioxidant peptide (Khan et al. 2013). The presence of alternating aromatic and cationic amino acids in the peptides could provide effective antioxidant properties and efficient membrane penetration (Szeto 2006). Thus, the amino acids constituent in a peptide determine its effectiveness. The supra-molecular system utilizes monolayer-protected gold nanoparticles (GNPs) so that the antioxidants and gold nanoparticles improve its functionality. This is consistent with the assertion that the organic molecules in organized assemblies having reactivity higher than the sum of the monomers. Au@Trolox, a gold nanoparticle with a Trolox (vitamin E analog)-derivative monolayer, possesses eight times higher radical scavenging activity compared with the Trolox monomer, indicating that the cooperative effect of supra-molecular assemblies also works in the area of free radical scavenging. Similarly, another study using Atm-deficient mice model confirmed that oxidative stress decreased after treatment of Atm-deficient mice with alpha-tocopherol (Erker et al. 2006).

### Cytotoxicity assessment of Pep-A and B

**Fig. 7:**
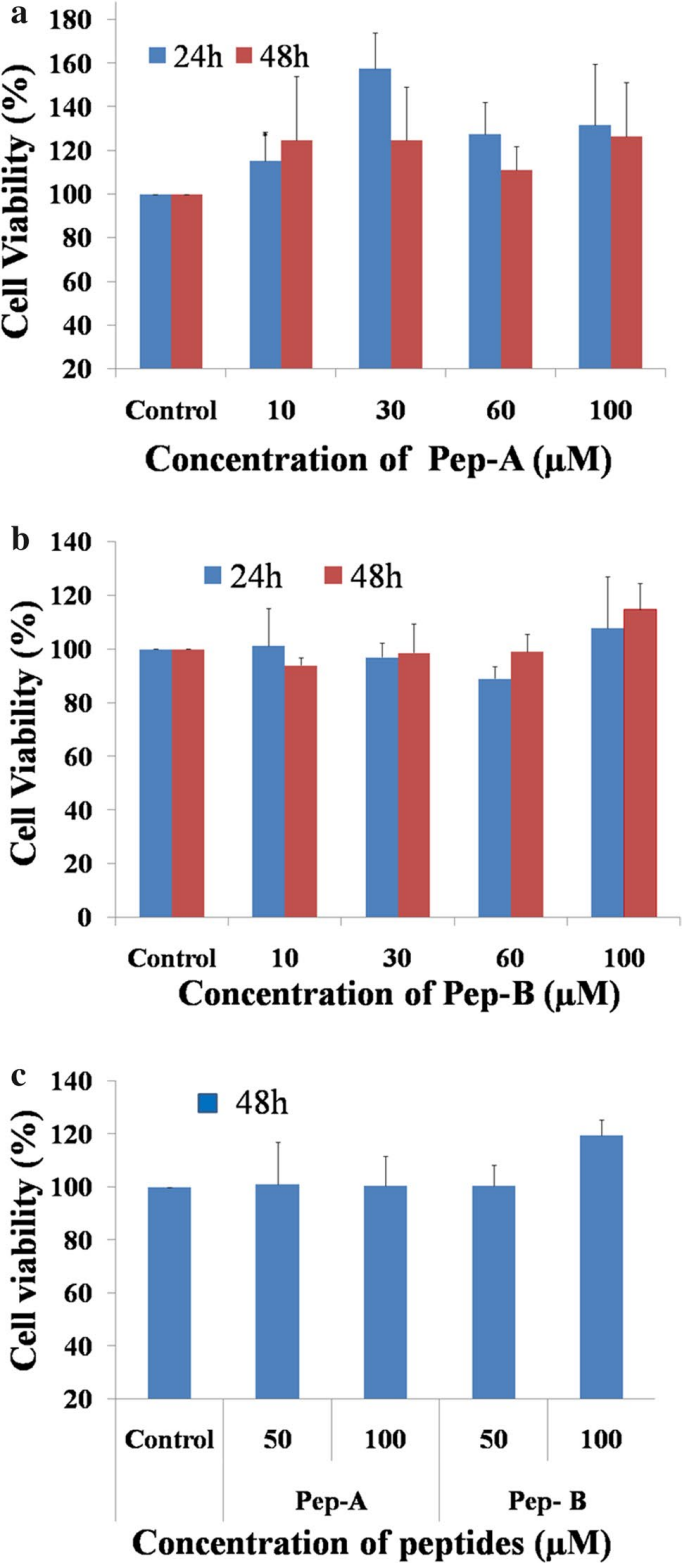
Cytotoxicity (MTT) assays with Pep-A and Pep-B. Cytotoxic effects of Peptide A and B analyzed at 24 and 48 h in Y79 RB cells (**a**) and (**b**), and MIO-M1 non-neoplastic cells (**c**), which were found to show no cytotoxicity in vitro

The GNP-Pep-A showed super performance as an antioxidant through ROS targeting compared to its constituents, Peptide A and GNPs. The 50-µM concentration of the GNPs shows more than 70 % of cell viability till 24 h in retinoblastoma cells; therefore, this concentration was considered for the in vitro functional studies. The above studies confirmed the GNPs-Pep-A stability and its uptake inside the Y79, RB cells (Fig. 5a). In addition to this, cell viability analysis results showed that the pep-A showed no toxicity to cancerous (Y79) cells and non-cancerous cells even after 48 h of treatment (Fig. 7a and c). The Y79 RB cell viability ranged between 115 and 157 % and 111–126 % after 24 and 48 h of exposures with Pep-A, respectively (Fig. 7a). The cancer cell death from the treatment of 10–100 µM GNPs concentration was studied (Fig. 7a). The 100 µM GNPs shows cell death on Y79 RB cells at 24 h of treatment, which could be partly due to the presence of polyphenols of GNPs. The earlier study reported with *V. vinifere* that nanoparticles were not toxic to breast cancer (MDA-MB-453) and non-neoplastic MIOM1 cells, indicating that the cytotoxicity of the nanoparticle could be cell-type specific (Kalmodia et al. 2013). The antiproliferative nature of GNPs in the retinoblastoma cell line could be due to the S-phase arrest leading to cell death in the presence of the polyphenols presented on the surface of GNPs.

The cancer cells are known to have elevated ROS and altered signaling mechanisms compared to normal cells. The elevated ROS in the cancer cells needs more efficient antioxidants to kill the cells. The GNP-Pep-A bioconjugate was a better antioxidant (Fig. 6) interacting with elevated ROS in the cancer cells causing changes in cell morphologies could be due to membrane blebbing, indicative of apoptosis leading to its death (Fig. 5). Antioxidant treatment strategies can increase hydrogen peroxide levels, scavenge reactive oxygen species (ROS), and induce cancer cells to undergo apoptosis (Gorrini et al. 2013; Trachootham et al. 2009). From, the experimental data, we could relate a similar mechanism operating in the death of cancer cells by the introduction of GNPs-Pep-A conjugate.

## Conclusion

Functionalized nanomaterials have revolutionized the field of targeted cancer therapy. Cancer biomarkers or targets such as onco-proteins are targeted with the functionalized nanomaterials. This study emphasizes on targeting the ROS in Retinoblastoma (RB) cancer cells, and also on an in vitro model to deliver the antioxidant peptides. The results have demonstrated an effective system for ROS scavenging activity which induces the apoptosis in RB cancer cells. The observed results confirm the synergistic effect of the GNPs-Pep-A. The novel, multifunctionalized GNPs can be alternative candidates for cancer therapeutic strategy —such as those demonstrated in case of current study—the self-therapeutic GNPs (such as *V. vinifrea L. *GNPs) and antioxidant peptides. Thus, the antioxidant-based therapy may improve response to the therapy, and therefore, in future, these novel GNPs-Pep-A can be promising nano biocomposites for targeting cancer cells. Although the application of antioxidants is controversial in cancer treatment, the effect of antioxidant can minimize the side effect of chemotherapy, which in turn, can reduce the chemical toxicity, and may, therefore, enhance the degree of success during the course of treatment.
